# The effect of a decision-support mHealth application on maternal and neonatal outcomes in two district hospitals in Rwanda: pre – post intervention study

**DOI:** 10.1186/s12884-022-04393-9

**Published:** 2022-01-20

**Authors:** Aurore Nishimwe, Latifat Ibisomi, Marc Nyssen, Daphney Nozizwe Conco

**Affiliations:** 1 School of Public Health, Faculty of Health Sciences, University of the Witwatersrand, 1 Smuts Avenue, Braamfontein, Johannesburg, 2000 South Africa; 2 School of Public Health / College of Medicine and Health Sciences, University of Rwanda, P.O. Box 3286, Kigali, Rwanda; 3 Nigerian Institute of Medical Research, 6 Edmund Cres, Yaba, Lagos, Nigeria; 4 Department of Biomedical Statistics and Informatics, Vrije Universiteit Brussel, Brussels, Belgium

**Keywords:** Maternal outcome, Neonatal outcome, BEmONC, mHealth, Safe delivery application, Rwanda

## Abstract

**Background:**

Globally, mobile health (mHealth) applications are known for their potential to improve healthcare providers’ access to relevant and reliable health information. Besides, electronic decision support tools, such as the Safe Delivery mHealth Application (SDA), may help to reduce clinical errors and to ensure quality care at the point of service delivery. The current study investigated the use of the SDA and its relationship to basic emergency obstetric and newborn care (BEmONC) outcomes for the most frequent complications in Rwanda; post-partum haemorrhage (PPH) and newborn asphyxia.

**Methods:**

The study adopted a pre–post intervention design. A pre-intervention record review of BEmONC outcomes: Apgar score and PPH progressions, was conducted for 6 months’ period (February 2019 - July 2019). The intervention took place in two district hospitals in Rwanda and entails the implementation of the SDA for 6 months (October 2019- March 2020), and included 54 nurses and midwives using the SDA to manage PPH and neonatal resuscitation. Six months’ post-SDA intervention, the effect of the SDA on BEmONC outcomes was evaluated. The study included 327 participants (114 cases of PPH and 213 cases of neonatal complications). The analysis compared the outcome variables between the baseline and the endline data. Fisher’s exact test was used to compare the proportions and test between-group differences and significance level set at *p* < 0.05.

**Results:**

Unstable newborn outcomes following neonatal resuscitation were recorded in 62% newborns cases at baseline and 28% newborns cases at endline, *P*-value = 0.000. Unstable maternal outcomes following PPH management were recorded in 19% maternal cases at baseline and 6% maternal cases at endline, *P*-value = 0.048. There was a significant association between the SDA intervention and newborns’ and maternal’ outcomes following neonatal resuscitation and PPH management, 6 months after baseline.

**Conclusion:**

The use of the SDA supported nurses and midwives in the management of PPH and neonatal resuscitation which may have contributed to improved maternal and neonatal outcomes during 6 months of the SDA intervention. The findings of this study are promising as they contribute to a broader knowledge about the effectiveness of SDA in low and middle income hospital settings.

## Background

Mhealth refers to the practice of medicine and public health, supported by mobile devices (mobile phones, smartphones, tablets, smartwatches) [1]. Evidence suggests that mHealth applications are innovative approaches to improve the delivery of health care services [2]. Further, mhealth tools can help to reduce clinical errors and ensure quality care at the point of service delivery. There is growing evidence of the effectiveness of mHealth interventions in maternal and newborn health programs in low and middle-income countries (LMICs), particularly to improve adherence to treatment, meeting appointments, facilitate data collection, and the development of supportive networks for health professionals [3–6].

Among the many interventions currently being implemented to support maternal and newborn healthcare services, mHealth applications have been widely used in low-resources settings [4, 7–9] as a potential solution to maximize health providers’ efficiency, health outcomes [9–11] and improve service utilization [2]. Common areas of application of mHealth tools include point-of-care decision-making support; provider-to-provider communication; and data collection [12, 13]. Though mHealth interventions are well received by healthcare providers [6, 14, 15] information about their effectiveness with regards to patient outcomes is limited [5, 9, 16].

Many interventions on the use of mHealth for improving maternal and newborns’ health outcomes in sub-Saharan Africa mainly focused on timely access to health facilities including reminders for antenatal appointments and referrals of mothers [17]. For instance, the CommCare mHealth technology is a digital solution that has shown promising results in assisting community health workers (CHWs) in data collection, decision support, communications with clients and health centres, and access to educational training materials [18]. Few mHealth applications are addressing the support of healthcare providers at the point of care with the main focus on clinical decision support in order to improve maternal and newborns outcomes [5, 9, 11, 19–22]. The Safe Delivery mHealth Application (SDA) is one of the recent mHealth applications (loaded in smartphones) which avails BEmONC clinical guidelines to support nurses and midwives’ clinical decisions [23].

Rwanda, a sub-Saharan African country, reports high maternal and neonatal deaths. In 2015, Rwanda’s maternal mortality was estimated at 290 deaths per 100,000 live births and its neonatal mortality rate was 20 deaths per 1000 live births, respectively [24] despite 90% of deliveries taking place in healthcare facilities attended by health professionals [25, 26]. Common causes of these deaths are preventable including postpartum hemorrhage (22.7% of all documented cases) and newborn asphyxia and its complications (38%) [27–29]. Challenges faced by healthcare providers in Rwanda include poor access to clinical guidelines and the lack of timely response to pregnancy complications during delivery care [30, 31]. It is in this context that a mHealth application - a clinical decision support tool (SDA) to facilitate easy access to maternal and neonatal guidelines for routine and emergency obstetric and neonatal care was introduced. The SDA has been tested for its effectiveness through a randomized controlled trial conducted in Ethiopia [32]. The SDA has also shown a positive effect on nurses and midwives’ knowledge and skills in the management of PPH and neonatal resuscitation in Rwanda [33]. This study investigated the use of the SDA and its relationship to BEmONC outcomes for the most frequent birth-related complications of PPH and newborn asphyxia in Rwanda. The current paper addresses one objective of a broad study about implementation of the SDA in Rwanda.

## Methods

### Study design

This was a pre–post intervention study done over a 14-month period. The study was conducted in three phases. The first phase was the pre-SDA intervention baseline study over a period of 6 months (February 2019 - July 2019). During this phase, a record review was conducted to document maternal and neonatal outcomes, PPH and Apgar score progressions, at baseline. The second phase, the SDA intervention (August 2019 - September 2019) comprised of the capacity building of nurses and midwives on the usage of the SDA; the SDA provision; the SDA piloting, and the launch of the SDA which marked the start of the SDA implementation. More details on the SDA training are documented in the protocol paper for this study [34]. The last phase was the 6 months of post-SDA intervention (October 2019- March 2020). Phase three encompassed the implementation of the SDA and another record review was conducted to document maternal and neonatal outcomes, PPH and Apgar score progressions, at endline. The data were collected from the delivery registries using data extraction forms. The collected data included patients’ characteristics and information on maternal and newborn outcomes, Apgar scores and PPH progressions, following NR and PPH management. The data were collected by the researcher and four research assistants. Data accuracy and transcription were checked by the researcher before analysis.

### Study setting

The study was conducted in two district hospitals in Rwanda: Masaka hospital in Kigali, an urban province; and Nyamata hospital located in the eastern rural province [26]. The two hospitals were chosen out of 12 district hospitals in the two provinces because both recorded a high number of deliveries per year [24]. Compared to other rural-based district hospitals, Nyamata reported the highest maternal and neonatal mortality rates [30].

### Study participants

**Fig. 1:**
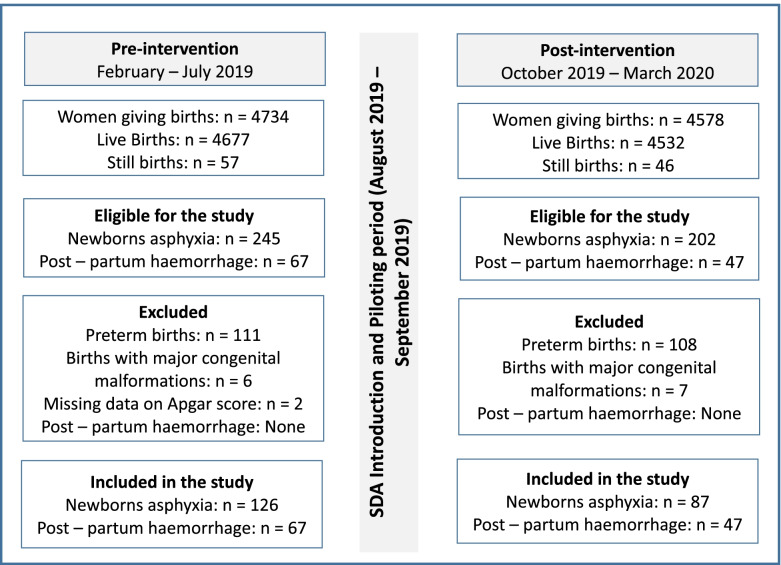
Records review flow chart

The study participants included records on neonatal complication cases and PPH cases. The inclusion criteria for the cases of neonatal complications were set as babies born with asphyxia (Apgar score ≤ 7) and were subject to neonatal resuscitation. We excluded preterm births (≤ 36 completed weeks of gestation) and births with major congenital malformations because prematurity and congenital complications other than the newborn asphyxia could influence the newborn outcome after resuscitation [35]. We have also excluded records with missing data on Apgar scores and neonatal outcome. On the other hand, the inclusion criteria for PPH cases were set as women who had a recorded amount of blood loss more than or equal to 500 mls and were subjected to PPH management. The records with missing data on PPH progression and maternal outcomes were also excluded. The study was conducted in three phases. Phase one, pre-intervention, included 126 cases of newborns asphyxia and 67 cases of PPH. Phase two, intervention, involved 33 midwives and 21 nurses who used the SDA in both Masaka and Nyamata district hospitals. Nurses and midwives were chosen because they are the frontline healthcare providers in childbirth care in district hospitals of Rwanda. The inclusion criteria for nurses and midwives were set as follows: having a work experience of at least 6 months in obstetric care and willing to participate in the study. All nurses and midwives (*n* = 54) working in the maternity departments of the selected hospitals volunteered to participate in the study and they were trained on the use of SDA. Phase three, post-intervention, comprised 87 cases of newborns asphyxia and 47 cases of PPH. The flowchart in Fig. 1 shows the number of neonatal complication cases and PPH cases considered in the pre-intervention group (before the introduction of SDA) and the post-intervention group (after SDA).

### Description of the intervention: implementation of the safe delivery mhealth application (SDA)

The SDA is a smartphone application developed by the Maternity Foundation, Copenhagen University, and the University of Southern Denmark. It is designed to support nurses and midwives in clinical decision making, by providing them with instant access to evidence-based BEmONC clinical guidelines and a selection of essential preventive protocols in a user-friendly format. The content of the SDA is primarily based on WHO (World health organization) clinical BEmONC guidelines and has been validated by an international group of global health experts [36]. The SDA contains easy to understand animated instruction videos, action cards with validated clinical guidelines, essential drugs lists with their indications, practical procedures guidelines, and a learning platform [32]. All features and functions in the SDA are designed for low-literacy and low-income settings and work offline once downloaded. The SDA can be used as a job aid and as an instructive aid in emergencies. While practicing, the skilled birth attendant (SBA) can consult action cards and drug lists to be helped in his/her clinical decision making. In the free time, the SBA can again play animated instruction videos, consult procedures descriptions and use the learning platform to update his/her knowledge and skills. The SDA can be downloaded free of charge for iPhone at https://itunes.apple.com/dk/app/safe-delivery/id985603707?mt=8 and for Android at https://play.google.com/store/apps/details?id=dk.maternity.safedelivery&hl=en.

The nurses and midwives in both hospitals received an explanation of the nature and purpose of the research and half-day training session on the use of the SDA. The majority of participants have downloaded the SDA on their personal smartphones during the training. The training session encompassed: an introduction to the research project; an overview of the SDA development and global outreach; description of the SDA features and modules; and hands-on practice on using the SDA as a job-aid and as a learning tool. Each of the study settings (Masaka and Nyamata) received three smartphones, with pre-installed English and French versions of the SDA. This was followed by the 6 months’ intervention period, during which nurses and midwives provided BEmONC services with the assistance of the SDA installed on their personal smartphones or the smartphones provided by the researcher. During this period, the provided six smartphones were made available to the team on duty (three staffs per shift per hospital) at all times for use as a backup in case the participants experienced problems with their own smartphones. Nurses and midwives were expected to use the SDA as often as they wished. The intervention also entailed two visits per week by two research assistants to each of the study hospitals. During the visits, the research assistants monitored the SDA use by nurses and midwives.

### Data collection

Data extraction forms were used to retrieve information on all cases of PPH and neonatal complications recorded in delivery registers. These were extracted and reviewed 6 months’ pre-SDA intervention and 6 months’ post-SDA intervention. The delivery registers contain information on obstetric and neonatal care including the Apgar scores and PPH progressions following neonatal resuscitation and PPH management. Newborn deaths or admission to the neonatal intensive care unit (NICU) or to neonatologist’s service due to an unstable outcome (Apgar score < 7) after neonatal resuscitation was used as the primary outcome measure to determine the failure of neonatal resuscitation. The mothers’ referral to a higher-level facility (referral hospital) following an unstable outcome (persistent bleeding) after PPH management was considered as the primary outcome to measure the failure of PPH management at the district hospital. On the newborn side, we obtained data on Apgar scores progression and neonatal outcome following 10 min’ neonatal resuscitation before and after the use of the SDA. While on the mother’s side, we obtained data on maternal outcomes following PPH management before and after the use of the SDA.

### Ethics approval

This study has been approved by the Human Research Ethics Committee of the University of the Witwatersrand (M190258) and the University of Rwanda, College of Medicine and Health Sciences’ Institutional Review Board (No.377/CMHS IRB/2018). Permission to collect data has been granted by the hospitals which have authorization of using aggregated patient data in research. Consents to track the SDA usage were obtained from nurses and midwives. This study was carried out in accordance with relevant guidelines and regulations in the Ethical Declarations.

### Statistical analysis

The data were checked for errors and exported from Microsoft Excel (Microsoft Corporation) to Stata version 16 (StataCorp LLC) for cleaning and analysis. Descriptive summary statistics were computed on data including the hospital, age of the mother at childbirth, the weight of the newborn at birth, sex of the newborn, mode of delivery, the leading clinician during delivery, Apgar scores at 1, 5 and 10 min and the resuscitation action taken for the newborns. For the maternal outcome, descriptive statistics included the hospital, age of the mother at delivery, gestational age in weeks, Mode of delivery, the leading clinician during delivery, Blood loss in milliliters, causes of PPH, the resuscitation action taken, mother outcome and cause of maternal death if dead. Further, the neonatal outcomes and maternal outcomes were compared at baseline and endline using Fisher’s exact test. Significance level was set at *p* < 0.05.

## Results

### Demographic characteristics of end-users of the SDA

In total, 54 healthcare providers, 21 nurses and 33 midwives used the SDA during the intervention period. More than a half of the participants were from Masaka district hospital (56%), the majority of them were female (61%), the participants had an average age of 33 years (SD = 7.1). In terms of their level of education, most participants hold an advanced diploma (A1) in midwifery (50%). Only one participant had the secondary school level (A2) in nursing. The majority of the nurses and midwives had less than 6 years of experience in the obstetric care (59%), spent more than 10 h per week providing services in obstetric care (74%), and participated in the care of more than ten deliveries per month (76%). Four participants acknowledged having never used a smartphone before the study. Additional details are shown in Table 1.

**Table 1:** The demographic characteristics of end-users of the SDA (*N* = 54)

	n (%)
**Hospital Affiliation**	
Masaka District Hospital	30 (56)
Nyamata District Hospital	24 (44)
**Sex**	
Male	21 (39)
Female	33 (61)
**Level of Education**	
Midwife A0	6 (11)
Midwife A1	27 (50)
Nurse A0	5 (9)
Nurse A1	15 (28)
Nurse A2	1 (2)
**Experience in obstetrical care (years)**	
1–5	32 (59)
6–10	15 (28)
> 10	7 (13)
**Weekly workload in obstetric care (hours)**	
0–5	4 (7)
6–10	10 (19)
> 10	40 (74)
**Number of deliveries past month**	
0–5	8 (15)
6–10	5 (9)
> 10	41 (76)
**Experience with the smartphone**	
Tried using one	50 (93)
Never tried using one	4 (7)
**Age, years, Mean (SD)**	33 (7.1)

*Abbreviations*: *%* Weighted percent, *SD* Standard deviation

### SDA usage tracking

The SDA usage by nurses and midwives was tracked through the server of Maternity Foundation (Table 2). We found that the most frequently accessed feature was the action cards, which was used 199 times and 128 times in the midterm (at 3 months’ intervention period) and endpoint (at 6 months’ intervention period) respectively. The duration of time spent in the action cards by users varied from 282 min at midterm to 371 min at endpoint. The action cards were used by 66 users at mid-term and 80 users at endpoint. The number of users is higher than the study sample (*n* = 54) because some participants have downloaded the SDA on their own smartphones in addition to the phones provided by the researcher and they were using both phones at different occasions. The least accessed feature was the essential drugs, which was used 26 times and 87 times in the midterm and endpoint respectively. The duration of time spent in the essential drugs by users varied from 14 min at midterm to 33 min at endpoint. The essential drugs were consulted by 14 participants at midterm and 23 participants at endpoint. For some features (action cards and essential drugs) the number of participants using the feature and the times spent in the SDA increased from midterm to endpoint. While for other features (practical procedures and videos), the number of participants using the feature and the session counts decreased from midterm to endpoint.

**Table 2:** SDA use by nurses and midwives

**SDA App Features**	**Midterm**		**End-Point**	
	Session Count	Session duration	Session Count	Session duration
	No. (number of times)	No. (duration in minutes)	No. (number of times)	No. (duration in minutes)
**Action Cards**	66 (199)	66 (282)	80 (128)	80 (371)
**Essential drugs**	14 (26)	14 (14)	23 (87)	23 (33)
**Practical Procedures**	37 (87)	37 (195)	17 (26)	17 (64)
**Videos**	53 (104)	53 (50)	33 (62)	32 (47)
**My Learning**	**Started the learning process, No. (%)**		**Passed the ‘MyLearning’ surveys, No. (%)**	
Familiar	44 (85)		48 (92)	
Proficient	43 (83)		40 (77)	
Expert	39 (75)		39 (75)	
**SDA champions**			**2 (3.85)**	
**Global Total SDA downloads_by March 2020, No. (%)**	126,444 (100)			
**Total SDA downloads in Rwanda_by March 2020, No. (%)**	179 (0.14)			

*Abbreviations*: *No* Number of content users, *%* Weighted percent

With regards to ‘MyLearning feature’, Table 2 shows that a good portion of study participants (*n* = 44, 85%) had started the familiar learning process in the midterm and by the endpoint 92% of the study participants had passed the familiar questionnaire. Similarly, a good portion of study participants (*n* = 43, 83%) had started the proficient learning process in the midterm and by the endpoint 73% of the study participants had passed the proficient questionnaire. Finally, at endpoint, 75% of the study participants had passed the expert questionnaire. At the end of the intervention period, we had two SDA champions. The maternity foundation’s server had also recorded 179 SDA users in Rwanda up to March 2020, meaning that our study participants in addition to downloading the SDA app on their own phones, they could have also shared the SDA to their colleagues in other hospitals apart from the hospitals included in the study. An exploration of the SDA acceptability was done at endline using surveys and focus group discussions with nurses and midwives. The findings of the SDA acceptability will be documented in a separate paper.

### Cases of newborns asphyxia

The analysis included 126 cases of newborns asphyxia in the baseline and 87 in endline groups over the study period. Table 3 shows that the majority of cases were from Masaka district hospital in the baseline group (55%) while in the endline group more cases were from Nyamata district hospital (56%). There were more male newborns for the baseline group (*n* = 79, 63%) and the endline group (*n* = 54, 62%). The most frequent mode of delivery was the spontaneous vaginal delivery in both the baseline group (*n* = 77, 61%) and the endline groups (*n* = 45, 52%). The leading clinicians during delivery, also in charge of neonatal resuscitation were mainly midwives (*n* = 71, 56%) in the baseline group and (*n* = 44, 51%) in the endline group. The average age of the mother was similar, 28 years and 27 years in the baseline and endline groups, respectively. The average weight of the newborn was similar, 2900 g, and 2800 g in the baseline and endline groups, respectively. The average Apgar score at birth was the same, 6/10 in both the baseline and endline groups. However, the average Apgar score after 10 min of resuscitation was lower in the baseline group (8/10) than the endline group (9/10). The resuscitation actions taken were mostly dominated by aspiration and ventilation with Ambu bag in both groups, baseline (82%), and endline (79%). The endline group had higher numbers of newborns with stable outcome after resuscitation (*n* = 64, 74%) than the baseline group (*n* = 40, 32%). Overall, the endline group had fewer newborns transferred to NICU or neonatology service (*n* = 24, 28%) than the baseline group (*n* = 78, 62%).

**Table 3:** Participants characteristics_ Newborns asphyxia (*N* = 213)

	Baseline n (%)	Endline n (%)
**Hospital Affiliation**		
Masaka District Hospital	69 (55)	38 (44)
Nyamata District Hospital	57 (45)	49 (56)
**Sex**		
Male	79 (63)	54 (62)
Female	47 (37)	38 (38)
**Mode of Delivery**		
Caesarean Section	49 (39)	42 (48)
Spontaneous Vaginal Delivery	77 (61)	45 (52)
**Leading Clinician of the Delivery**		
Doctor	49 (39)	42 (48)
Midwife	71 (56)	44 (51)
Nurse	6 (5)	1 (1)
**Resuscitation Actions**		
Aspiration, Ventilation with Ambu bag	103 (82)	69 (79)
Aspiration, Ventilation, Oxgenotherapy	12 (9)	6 (7)
CPR, Ventilation, Oxygenation	11 (9)	12 (14)
**Outcome after 10 min Resuscitation**		
Transferred to NICU or Neonatology Service	78 (62)	24 (28)
Skin to skin bonding with mother	39 (31)	63 (72)
Dead	9 (7)	0 (0)
**Weight of the newborn, grams, Median (IQR)**	2900(880)	2800(800)
**Mother’s age, years, Median (IQR)**	28(7)	27(7)
**Apgar Scores, Median (IQR)**		
At 1 min	6(2)	6(2)
At 5 min	7(2)	7(1)
At 10 min	8(2)	9(2)

*Abbreviations*: *%* Weighted percent, *IQR* Interquartile range

### Cases of postpartum haemorrhage (PPH)

The analysis included 114 cases of Post-partum haemorrhage (PPH): 67 in baseline and 47 in endline groups for both Masaka and Nyamata district hospitals over the study period. Table 4 shows that the majority of cases were from Masaka district hospital in the baseline group (54%) while in the endline group more cases were from Nyamata district hospital (55%). The most frequent mode of delivery was the spontaneous vaginal delivery in both the baseline group (*n* = 47, 70%) and the endline groups (*n* = 34, 72%). The leading clinicians of the delivery, also in charge of PPH management were mainly midwives (*n* = 36, 54%) in the baseline group and (*n* = 25, 53%) in the endline group. The average age of the mother was similar, 28 years in the baseline and endline groups, respectively. The average gestational age was similar, 39 weeks in the baseline and endline groups, respectively. The mean estimated amount of blood loss was 1167.2 mls in the baseline group and 1178.7 mls in the endline group. The most frequent cause of PPH was the uterine atony in both the baseline (72%) and the endline group (70%). The resuscitation actions taken were mostly dominated by administering oxytocin, IV fluids, and blood transfusion in both groups, baseline (37%), and endline (55%). The endline group had higher numbers of women with stable outcomes after PPH management (*n* = 44, 94%) than the baseline group (*n* = 55, 78%). Overall, the endline group had fewer women transferred to referral hospitals after PPH management (*n* = 3, 6%) than the baseline group (*n* = 13, 19%).

**Table 4:** Participants characteristics_ PPH (*N* = 114)

	Baseline n (%)	Endline n (%)
**Hospital Affiliation**		
Masaka District Hospital	36 (54)	21 (45)
Nyamata District Hospital	31 (46)	26 (55)
**Mode of Delivery**		
Caesarean Section	20 (30)	13 (28)
Spontaneous Vaginal Delivery	47 (70)	34 (72)
**Leading Clinicians of the Delivery**		
Doctor	20 (30)	13 (28)
Midwife	36 (54)	25 (53)
Nurse	11 (16)	9 (19)
**Causes of PPH**		
Uterine atony	48 (72)	33 (70)
Cervical tear	11 (16)	7 (15)
Retention of Placenta	8 (12)	7 (15)
**Resuscitation Actions**		
Oxytocin, IV fluids	23 (34)	7 (15)
Oxytocin, IV fluids, Transfusion	25 (38)	26 (55)
Repair of tear, IV fluids	11 (16)	7 (15)
Removal of Placenta, IV fluids	8 (12)	7 (15)
**Maternal outcome after PPH Management**		
Transferred to Referral Hospital	13 (19)	3 (6)
Stable	52 (78)	44 (94)
Dead	2 (3)	0 (0)
**Mother’s age, years, Median(IQR)**	28(5)	28(5)
**Gestational age, weeks, Median(IQR)**	39(0)	39(0)
**Blood loss in Mls, Mean (SD)**	1167.2 (276.6)	1178.7 (257.1)

*Abbreviations*: *%* Weighted percent, *IQR* Interquartile range, *SD* Standard deviation

### Pre-post differences in newborns outcomes following neonatal resuscitation

We found a statistically significant association between the SDA intervention and newborns’ outcomes following neonatal resuscitation, 6 months after baseline (Table 5). Among 213 cases of newborns complications who were included in the study, stable outcomes following neonatal resuscitation were recorded in 31% of newborns cases at baseline and 72% at endline. The transferred (unstable) newborns were 62 and 28% at baseline and endline respectively. While death following neonatal resuscitation appeared in 7% for the baseline group only. No death was recorded in the endline group. There was a significant difference between baseline and endline in the outcome (stable, transferred, and dead) for newborns who underwent neonatal resuscitation (*p* = 0.000, Fisher’s exact test).

**Table 5:** Newborns outcomes following neonatal resuscitation (*N* = 213)

	Before SDA (*N* = 126), n(Wt.%)	After SDA (*N* = 87), n(Wt.%)	*P*-Value (Fisher’ exact test)
**Newborns Outcome**			
Stable	39 (31)	63 (72)	*P*(Fisher) = 0.000
Unstable(Transferred)	78 (62)	24 (28)	
Dead	9 (7)	0 (0)	

*Abbreviations*: *Wt.%* Weighted percent

### Pre-post differences in maternal outcomes following PPH management

We found a statistically significant association between the SDA intervention and maternal’ outcomes following PPH management, 6 months after baseline (Table 6). Among 114 cases of PPH who took part in the study, stable outcomes following PPH management were recorded in 78% PPH cases at baseline and 94% at endline. Transferred (unstable) women were 19 and 6% at baseline and endline respectively. While death attributed to PPH appeared in 3% women for the baseline group only. No death was recorded in the endline group. There was a significant difference between baseline and endline in the maternal outcome (stable, transferred, and dead) following PPH management (*p* = 0.048, Fisher’s exact test).

**Table 6:** Maternal outcomes following PPH management (*N* = 114)

	Before SDA (*N* = 67), n(Wt.%)	After SDA (*N* = 47), n(Wt.%)	*P*-Value (Fisher’ exact test)
**Maternal outcome**			
Stable	52 (78)	44 (94)	*P*(Fisher) = 0.048
Unstable(Transferred)	13(19)	3 (6)	
Dead	2 (3)	0 (0)	

*Abbreviations*: *Wt.%* Weighted percent

## Discussion

### Main findings

The current study investigated the use of the SDA and its relationship to BEmONC outcomes for the most frequent birth-related complications in Rwanda; PPH and newborn asphyxia. Apgar scores and PPH progressions were considered in the investigation of newborn and maternal outcomes following neonatal resuscitation and PPH management.

The findings from this study are consistent with SDA having the potential to improve clinical outcomes. These results are encouraging: nurses and midwives were willing to use SDA and the intervention was engaged with throughout the intervention period. Clinical data show a correlation between SDA implementation and improved maternal and newborn outcomes. However, the improved maternal and newborn outcomes may have been influenced by other factors in addition to the SDA intervention. Nevertheless, the findings of this study contribute to the broader knowledge about the SDA effectiveness and provide promising evidence to support the need for more rigorous and expensive research on the SDA.

### Comparison with other studies

As was noted in the present study, Lund et al. [32] also reported a significant effect of the use of the SDA particularly on skilled birth attendants in terms of significantly increased knowledge and skills scores in neonatal resuscitation in Ethiopia [32]. Our previous study also found a significant association between the SDA uptake/use and change in knowledge and skills of nurses and midwives in PPH management and NR [33]. Another study conducted in DRC Congo to determine the feasibility, acceptability, and potential effect of the SDA on health workers’ practices in BEmONC reported a significant increase of health worker knowledge and self-confidence in the management of obstetric and newborn emergencies after 3 months SDA intervention [37]. In addition to the previous studies on the SDA, the present study provided evidence on the SDA implementation, use, and clinical outcomes. However, the implicit theory of change for the SDA may follow a logical sequence of (a) the SDA implemented and used; (b) the use of SDA supports skilled birth attendants’ continuous learning and decision making; the use of the SDA changes clinical response behaviors and practices; (c) the changes in practices leads to improved clinical outcomes. Therefore, future research using a theory of change or a logical framework over a long period is needed to understand the dynamics and change processes of skilled birth attendants’ clinical practices when using the SDA.

Moreover, mHealth programmes have shown promising results for newborn outcomes. A study which looked at antenatal messaging services using a mobile phone intervention in Zanzibar reported a significant reduction in perinatal mortality [38]. This could be explained by the fact that mhealth applications have the potentiality to put relevant and reliable healthcare information into the hands of healthcare workers thus helping them in the management of maternal and neonatal cases. But also, that health professionals need continuous access to updated healthcare information and clinical guidelines to support their decision making during practices. The same opinion was reported in a similar study done by Grol et al. [39] about the effective implementation of mHealth interventions [39]. However, Tamrat et al. [40] in their systematic review which analysed mHealth interventions in maternal and newborn health programs and their outcomes around the world reported that few trials have been conducted and most maternal mHealth programmes have not been evaluated, and the evidence is still weak [40]. Thus the need for more evidence on maternal and newborn mHealth programmes.

Maternal outcome following PPH management was another significant predictor in the present study. Our findings are in agreement with other studies that documented mHealth interventions which have been tested in a variety of health services delivery including decision support and clinical management [41–43]. These studies found promising findings for the mhealth technology in general and for maternal healthcare services in particular. Then again, other studies have shown that the evidence of maternal mHealth interventions is supportive but weak. A 2017 systematic review on mHealth interventions in low-income countries demonstrated good promise for the use of mHealth interventions in maternal health, but the evidence is limited [20]. This review suggested more research on the relationship between mHealth and clinical outcomes because the majority of mHealth trials focused on very distinct mhealth applications (i.e appointment reminders and data collection). Another review which identified 51 RCTs, documented that nearly half of the reviewed RCTs (*n* = 22) showed negative or unclear results, thus the need for better evidence; and for caution with new interventions [44]. Another study which assessed the impact of the SDA on the incidence of PPH in Ghana reported that the SDA was associated with an insignificant lower incidence of PPH [45]. Hence, the need for more evidence and higher-quality research in this field. The current study adds to much-needed evidence as to how a decision-support mHealth application functioned in a low-resource setting. Future research that analyze event-event correlation between SDA use and maternal and newborn outcomes would be important.

## Limitations

Though our study documents important patterns of use of a mHealth to inform clinical decisions, the quantity of information accessed for the care of individual patients is limited in the current study. We acknowledge the limitations of our study design, pre-post intervention, which partly permitted us to demonstrate the effects of the SDA on maternal and newborn outcomes. The observed maternal and newborn outcomes may have been influenced by other factors in addition to the SDA intervention. Also, nurses and midwives may have consulted other sources rather than the SDA to inform their decision-making during the intervention. Although this limitation is inherent in the design of the study, the current study contributes to the broader evidence about the effectiveness of the SDA and provides much-needed insights as to how a decision-support mHealth application functioned in a low-resource setting.

## Conclusion

The use of the SDA supported nurses and midwives in the management of PPH and neonatal resuscitation which may have contributed to improved maternal and neonatal outcomes during 6 months of the SDA intervention. Mhealth interventions that focus on the clinical decision support process in maternal and newborn care may contribute to improved services delivery and should be considered by policymakers in resource-limited settings, like Rwanda. Overall, there is limited evidence on the effects of mHealth interventions on clinical outcomes on a large scale and further research is necessary to draw holistic conclusions, particularly for developing countries within the field of maternal and newborn care.

## Data Availability

The dataset generated for this study will be made available from the corresponding author on a reasonable request.

## Abbreviations

BEmONC: Basic Emergency Obstetric and Newborn Care
CHWs: Community health workers
CMHS IRB: College of Medicine and Health Sciences Institutional Review Board
IQR: Interquartile range
IV: Intravenous line
LMICs: Low- and middle-income countries
mHealth: Mobile Health
Mls: Millilitres
NICU: Neonatal intensive care unit
SDA: Safe Delivery mhealth Application
SD: Standard deviation
PPH: Post-Partum Hemorrhage
NR: Neonatal Resuscitation
SBA: Skilled Birth Attendants
WHO: World Health Organisation
